# Effectiveness and Safety of Once‐Weekly Semaglutide 2.4 mg in Korean Adults With Obesity or Overweight: A Multicenter Retrospective Real‐World Study (RE‐SEMA‐K Study)

**DOI:** 10.1002/dmrr.70242

**Published:** 2026-11-09

**Authors:** Yu‐Jin Kwon, Young‐Sang Kim, Hae‐Jin Ko, Susie Jung, Yoon Jeong Cho, Ji‐Eun Song, Sung‐Bum Lee, Ga Eun Nam, Jungha Park, Myung Jae Seo, Jong Koo Kim, Seo Young Kang, Yeong Sook Yoon, Soo Hyun Cho, Yonghwan Kim, Hye‐ji An, Seung Hee Kim, Yoo‐Bin Seo, Yong Ho Lee, Seok‐Jae Heo, Jee‐Hyun Kang, Kyoung‐Kon Kim

**Affiliations:** ^1^ Department of Family Medicine Yongin Severance Hospital Yonsei University College of Medicine Yongin Republic of Korea; ^2^ Department of Family Medicine CHA Bundang Medical Center CHA University Seongnam Republic of Korea; ^3^ Department of Family Medicine School of Medicine Kyungpook National University Kyungpook National University Hospital Daegu Republic of Korea; ^4^ Department of Family Practice and Community Health Ajou University School of Medicine Suwon Republic of Korea; ^5^ Department of Family Medicine Daegu Catholic University School of Medicine Daegu Republic of Korea; ^6^ Department of Family Medicine School of Medicine Kyungpook National University, Kyungpook National University Chilgok Hospital Daegu Republic of Korea; ^7^ Department of Family Medicine Soonchunhyang University Bucheon Hospital Bucheon Republic of Korea; ^8^ Department of Family Medicine Korea University Guro Hospital Korea University College of Medicine Seoul Republic of Korea; ^9^ Department of Family Medicine Kyung Hee University Medical Center Seoul Republic of Korea; ^10^ Department of Family Medicine Wonju Severance Christian Hospital Yonsei University Wonju College of Medicine Wonju Republic of Korea; ^11^ Department of Family Medicine Uijeongbu Eulji Medical Center Eulji University School of Medicine Uijeongbu Republic of Korea; ^12^ Department of Family Medicine Ilsan Paik Hospital Inje University College of Medicine Goyang Republic of Korea; ^13^ Department of Family Medicine College of Medicine, Chung‐Ang University Seoul Republic of Korea; ^14^ Department of Family Medicine Hallym University Sacred Heart Hospital Hallym University Anyang Republic of Korea; ^15^ Department of Family Medicine Wonkwang University Sanbon Hospital Gunpo Republic of Korea; ^16^ Department of Biomedical Systems Informatics Yonsei University College of Medicine Seoul Republic of Korea; ^17^ Department of Family Medicine Konyang University College of Medicine Konyang University Hospital Daejeon Republic of Korea; ^18^ Department of Family Medicine Gachon University College of Medicine Gachon University Gil Medical Center Incheon Republic of Korea

**Keywords:** obesity, overweight, real‐world study, semaglutide, South Korea

## Abstract

**Aims:**

To evaluate the effectiveness and treatment patterns of once‐weekly semaglutide 2.4 mg in South Korean adults with overweight or obesity in real‐world practice.

**Materials and Methods:**

This multicenter retrospective study analysed electronic medical records from 17 centres in South Korea. Included were adults (≥ 19 years) with BMI ≥ 25 kg/m^2^, or ≥ 23 kg/m^2^ with obesity‐related comorbidities. Primary endpoints were changes in body weight at 3, 6, 9, and 12 months. Secondary outcomes included body composition, categorical weight loss, cardiometabolic changes, treatment persistence, safety, and changes in liver function.

**Results:**

Among 1160 patients (40.9% with prior obesity medications), semaglutide treatment induced progressive weight reduction (−5.85%, −9.55%, −11.43%, and −13.16% at 3, 6, 9, and 12 months). Greater reduction occurred in women, younger patients, and those without T2D or prior obesity medication. By 6 months, cardiometabolic, liver function, and body composition parameters improved, with preferential loss of fat over lean mass (all *p* < 0.001). Starting doses varied across patients (0.25 mg, 55.9%; 0.5 mg, 30.4%; 1.0 mg, 11.9%), with rapid dose escalation reaching the maintenance dose (2.4 mg) in a mean of 3.52 months, reflecting individualised real‐world dosing. Mean treatment duration was 4.9 months. Adverse events, predominantly transient gastrointestinal symptoms, occurred in approximately 22% of patients.

**Conclusions:**

Semaglutide 2.4 mg achieved substantial weight loss in South Korean adults with overweight or obesity. Weight reduction was comparable to the STEP 6 trial despite shorter follow‐up (4.9 months), substantial prior obesity medication exposure, and accelerated dose escalation, supporting robust real‐world effectiveness.

## 1 Introduction

Obesity is a complex, chronic, and relapsing multisystem disease that has reached pandemic proportions and imposes considerable burdens on global health systems [1]. It is characterised by excess adiposity and is associated with a broad spectrum of metabolic, mechanical, and psychological complications, including type 2 diabetes (T2D), hypertension, dyslipidemia, metabolic dysfunction–associated steatotic liver disease (MASLD), and cardiovascular disease—the leading cause of mortality worldwide [1, 2].

The pathophysiology of obesity involves a complex interplay among genetic, environmental, and neuroendocrine factors, with hypothalamic regulation of appetite and energy balance mediated through the gut–brain axis playing a central role [2, 3]. Glucagon‐like peptide‐1 (GLP‐1), an incretin hormone secreted by intestinal L‐cells, enhances glucose‐dependent insulin secretion, suppresses glucagon release, delays gastric emptying, and promotes satiety [3, 4].

Semaglutide, a long‐acting GLP‐1 receptor agonist with a half‐life of ∼1 week, enables once‐weekly administration and has demonstrated substantial weight reduction in randomized clinical trials [5, 6, 7]. The Semaglutide Treatment Effect in People with Obesity (STEP) programme established the efficacy of semaglutide, with a mean weight loss of 14.9% at 68 weeks in STEP 1 and consistent findings in East Asian populations in STEP 6 and 11 [5, 6, 8].

Despite these robust trial data, notable gaps remain between efficacy in controlled settings and effectiveness in routine clinical practice. Clinical trials typically stratify participants by T2D status to reduce heterogeneity; however, real‐world populations are inherently mixed, and patients with T2D achieve less weight loss than those without diabetes, underscoring the importance of evaluating outcomes in more representative populations [5, 9, 10]. In routine clinical practice, many patients are not treatment‐naïve but transition from prior obesity medications, including liraglutide or phentermine/topiramate, patterns not fully captured in pivotal randomized trials [11]. Economic considerations further widen this gap [12]. In South Korea, where GLP‐1 receptor agonist reimbursement remains limited, out‐of‐pocket costs may substantially affect treatment initiation and persistence, highlighting the need for real‐world evidence in this setting.

Accordingly, we aimed to evaluate the effectiveness and treatment patterns of once‐weekly semaglutide 2.4 mg in a multicenter retrospective cohort of South Korean adults.

## 2 Materials and Methods

### 2.1 Study Design and Population

This multicenter, retrospective cohort study utilised electronic medical record (EMR) data from 17 academic and community‐based medical centres in South Korea. The study protocol was reviewed and approved by the Institutional Review Board of Yongin Severance Hospital, Yonsei University College of Medicine (lead IRB) (IRB No. 9‐2025‐0134), with additional approvals obtained from the Institutional Review Boards of all participating centres. The requirement for informed consent was waived owing to the study's retrospective nature and use of de‐identified data. This study was conducted in accordance with the ethical principles of the Declaration of Helsinki and its subsequent amendments.

We included adults aged ≥ 19 years who were prescribed once‐weekly subcutaneous semaglutide 2.4 mg between October 1, 2024, and December 31, 2025. Eligible participants had a body mass index (BMI) ≥ 25 kg/m^2^, or BMI ≥ 23 kg/m^2^ with at least one obesity‐related comorbidity (e.g., T2D, hypertension, dyslipidemia, or MASLD), in accordance with Asian‐specific obesity criteria and the South Korean Society for the Study of Obesity guidelines [13]. Additional inclusion criteria were availability of baseline weight measurement data within ± 30 days of treatment initiation and at least one follow‐up weight measurement during the study period.

### 2.2 Data Collection

Data were extracted from the EMR using a unified case report form. Collected variables included demographic characteristics, lifestyle factors, medical history, prior use of obesity medications, anthropometric measures, body composition, and laboratory parameters. Medical history encompassed cardiometabolic conditions, liver and kidney disease, obstructive sleep apnoea, malignancy, and psychiatric disorders, identified from physician‐documented diagnoses. Prior obesity medication use within 6 months before semaglutide initiation (e.g., liraglutide, phentermine/topiramate, naltrexone/bupropion, or orlistat) was recorded. Anthropometric measurements included body weight, height, BMI, and waist circumference (WC), defined according to Asian‐specific criteria and the South Korean Society for the Study of Obesity guidelines [14]. Body composition (fat and lean mass) was assessed by bioelectrical impedance analysis (BIA). Cardiometabolic parameters included blood pressure, glycated haemoglobin (HbA1c), fasting glucose, lipid profile, liver enzymes, and renal function. Baseline and follow‐up measurements were obtained at monthly intervals (± 14 days) for up to 12 months. Data were anonymised by site investigators to ensure patient confidentiality.

### 2.3 Study Outcomes

The primary outcome was percentage change in body weight from baseline to months 3, 6, 9, and 12. Secondary outcomes included changes in body composition, treatment persistence (including duration of use and reasons for discontinuation), safety and tolerability, changes in cardiometabolic risk factors, and changes in liver function parameters. In addition, the proportion of patients achieving clinically meaningful weight loss thresholds (≥ 5%, ≥ 10%, ≥ 15%, and ≥ 20% from baseline) was evaluated.

### 2.4 Statistical Analysis

Baseline characteristics were summarised using descriptive statistics. Continuous variables are presented as mean ± standard deviations, and categorical variables as frequencies and percentages.

Longitudinal changes in outcomes were analysed using generalised linear mixed‐effects models with random intercepts. Time was modelled as a continuous variable using restricted cubic splines with three knots to allow for flexible, nonlinear trajectories over follow‐up. A Gaussian identity link was used for continuous outcomes and a logit link for binary outcomes. All models were adjusted for the baseline value of the respective outcomes. Estimated marginal means were derived across the follow‐up period to characterise overall longitudinal trajectories and were estimated at 3, 6, 9, and 12 months with 95% confidence intervals (CI).

Subgroup analyses were performed according to baseline characteristics, including age, sex, BMI category, T2D status, and prior obesity medication use (including type of agent used). Interaction terms were included to assess the heterogeneity of treatment effects across the subgroups. To ensure the stability and reliability of estimates and minimise small‐sample bias, analyses were restricted to subgroups with at least 15 events, consistent with established recommendations regarding minimum event counts in regression analyses [15, 16].

### 2.5 Sample Size Consideration

The target sample size was determined based on prior real‐world data [17], assuming 80% statistical power and a two‐sided *α* of 0.05. Accounting for expected attrition, a minimum 1193 participants were planned for enrolment. The final analytic cohort of 1160 patients provided adequate statistical power for observed outcomes.

## 3 Results

### 3.1 Baseline Characteristics of the Participants

A total of 1160 patients (mean age: 44.5 ± 12.5 years; female, 61.4%) were included in the analysis. The mean baseline BMI was 32.4 ± 6.1 kg/m^2^, with 62.0% of patients presenting with a BMI ≥ 30 kg/m^2^. The mean body weight was 90.0 ± 21.8 kg, and the mean WC was 107.0 ± 39.7 cm. Most participants were South Korean (97.2%). Overall, 40.9% of the patients had received obesity medications within 6 months prior to semaglutide initiation. All baseline characteristics are presented in Table 1.

**TABLE 1:** Baseline characteristics of the participants (*N* = 1160).

Characteristic	Value
Age, years	44.5 ± 12.5
BMI, kg/m^2^	32.4 ± 6.1
BMI category, kg/m^2^, *n* (%)	
23–< 25	57 (4.9)
25–< 30	384 (33.1)
≥ 30	719 (62.0)
Weight, kg	90.0 ± 21.8
Waist circumference, cm	107.0 ± 39.7
Sex, *n* (%)	
Female	712 (61.4)
Ethnicity, *n* (%)	
Korean	1127 (97.2)
Alcohol use, *n* (%)	
Non‐drinker	455 (46.1)
Moderate drinking	459 (46.5)
Risky drinking	73 (7.4)
Smoking status, *n* (%)	
Never smoker	742 (75.3)
Former smoker	91 (9.2)
Current smoker	153 (15.5)
Comorbidities, *n* (%)	
Hypertension	431 (37.2)
Type 2 diabetes	206 (17.8)
Dyslipidemia	466 (40.2)
Liver disease	205 (17.7)
Cardiovascular disease	74 (6.4)
PHQ‐9 score	5.0 (0.0–27.0)
Previous anti‐obesity medication use, *n* (%)	
None	686 (59.1)
Liraglutide	290 (25.0)
Phentermine/topiramate	118 (10.2)
Naltrexone/bupropion	15 (1.3)
Orlistat	13 (1.1)
Others^a^	38 (3.3)
Metabolic parameters, mean ± SD	
Systolic blood pressure, mmHg	133.5 ± 17.8
Diastolic blood pressure, mmHg	82.1 ± 27.9
Fasting glucose, mg/dL	108.5 ± 27.5
HbA1c, %	6.07 ± 1.03
Total cholesterol, mg/dL	186.0 ± 45.3
LDL cholesterol, mg/dL	114.1 ± 41.1
HDL cholesterol, mg/dL	51.9 ± 13.3
Triglycerides, mg/dL	162.0 ± 97.4
AST, U/L	30.9 ± 21.4
ALT, U/L	38.4 ± 33.1

*Note:* Values are presented as mean ± standard deviations for continuous variables and number (percentage) for categorical variables. Several variables had substantial missing data. Waist circumference was missing in 648 participants (55.9%), Patient Health Questionnaire‐9 (PHQ‐9) scores in 724 (62.4%), systolic and diastolic blood pressure in 200 (17.2%); fasting glucose in 457 (39.4%); HbA1c in 557 (48.0%); total cholesterol in 606 (52.2%); LDL cholesterol in 517 (44.6%); HDL cholesterol in 521 (44.9%); triglycerides in 669 (57.7%); and AST and ALT in 442 (38.1%) each, and multiple lifestyle‐related variables—including physical activity, dietary patterns, and binge eating behaviour—in 25%–70%.

^a^ Others include fluoxetine, glucagon‐like peptide‐1 (GLP‐1) receptor agonists other than semaglutide (e.g., dulaglutide), prior semaglutide use (e.g., Ozempic), topiramate, bariatric surgery, and herbal medicine.

### 3.2 Weight Loss Effectiveness

Treatment with semaglutide 2.4 mg resulted in a continuous and progressive reduction in body weight over the 12‐month follow‐up period (Figure 1A). The estimated mean percentage change in body weight from baseline was −5.85% (95% CI, −6.13 to −5.58) at 3 months (*n* = 858), −9.55% (−9.84 to −9.26) at 6 months (*n* = 422), −11.43% (−11.77 to −11.09) at 9 months (*n* = 147), and −13.16% (−13.63 to −12.68) at 12 months (*n* = 23) (all *p* < 0.001) (Figure 1B). The proportion of patients achieving clinically meaningful weight loss increased over time. At 6 months, 78.1% achieved ≥ 5% weight loss and 50.0% achieved ≥ 10% weight loss. By 12 months, 29.3% achieved ≥ 15% weight loss and 14.6% achieved ≥ 20% weight loss (Supporting Information S1: Table 1).

**FIGURE 1:**
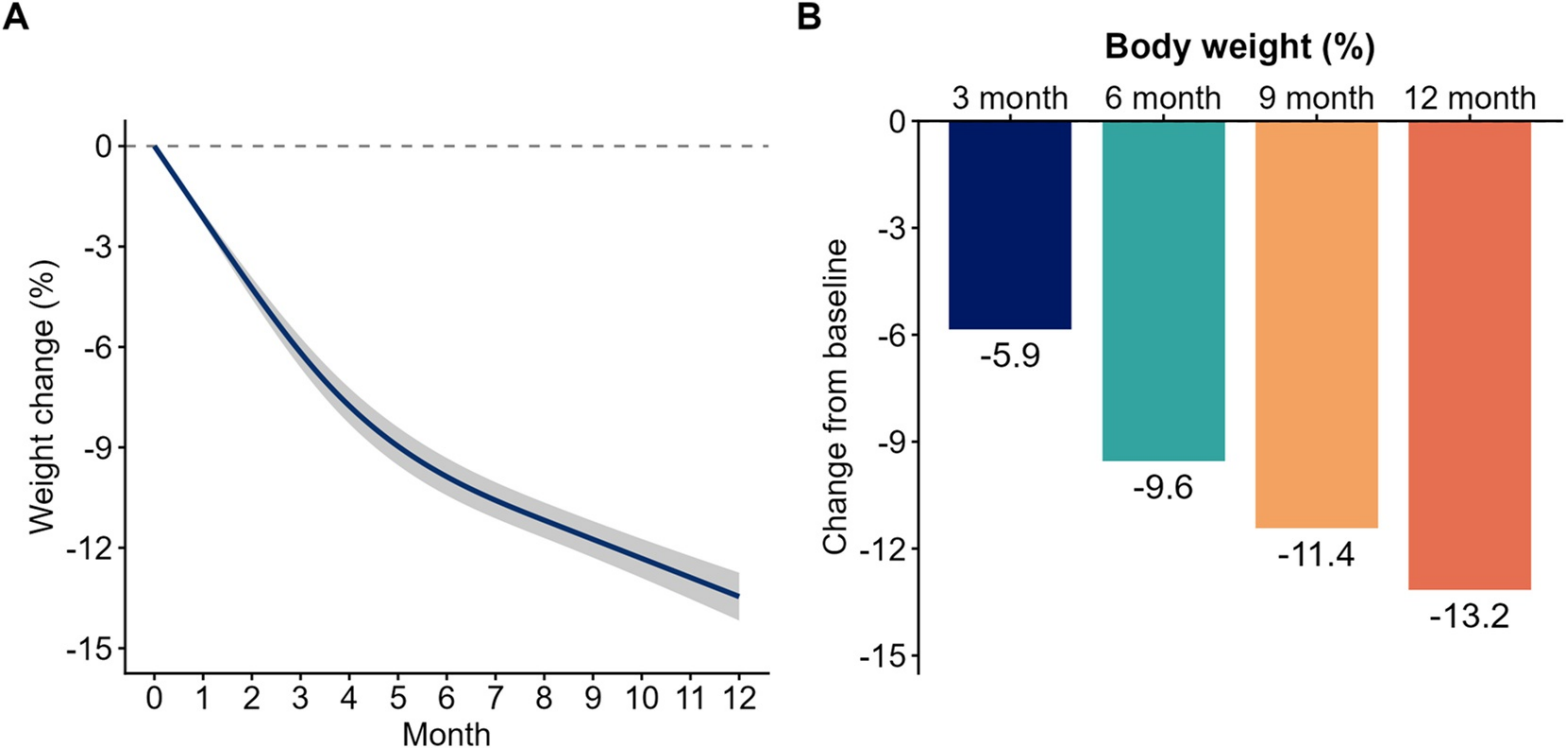
Percentage total body weight reduction over 12 months with semaglutide 2.4 mg (A) Estimated mean percentage change in body weight from baseline over time. (B) Estimated mean percentage change in body weight (%) at 3, 6, 9, and 12 months. Data in panel A are presented as estimated marginal means with 95% confidence intervals (shaded area) derived from a linear mixed‐effects model for repeated measures. Panel B shows observed mean changes at designated time points using data from the same model. The number of patients with available body weight measurements at each time was as follows: baseline (*n* = 1160), 1 month (*n* = 1100), 2 months (*n* = 984), 3 months (*n* = 858), 4 months (*n* = 709), 5 months (*n* = 565), 6 months (*n* = 422), 7 months (*n* = 302), 8 months (*n* = 199), 9 months (*n* = 147), 10 months (*n* = 89), 11 months (*n* = 47), and 12 months (*n* = 23).

### 3.3 Subgroup Analyses According to Baseline Characteristics and Prior Obesity Medication Exposure

Significant reductions in percentage body weight were observed across all BMI, age, sex, and T2D subgroups throughout the 12‐month follow‐up period (Figure 2A–D), with significant interactions detected for all four characteristics (BMI: *p* for interaction = 0.011; age, sex, and T2D: all *p* for interaction < 0.001). Despite the significant interaction, pairwise comparisons between BMI groups revealed no statistically significant differences in percentage body weight loss at any follow‐up time point, indicating consistent relative weight reduction regardless of baseline BMI. In contrast, participants aged < 40 years achieved significantly greater percentage body weight loss than those aged 40–59 and ≥ 60 years from 3 months onward (all *p* < 0.01), with no significant difference between the two older groups. Women demonstrated significantly greater percentage body weight loss than men from 6 months onward, with the difference increasing progressively over time (6 months: 1.36%; 9 months: 2.82%; 12 months: 4.33%; all *p* ≤ 0.01). Similarly, participants without T2D achieved significantly greater percentage body weight loss than those with T2D from 3 months onward, with the difference widening progressively (3 months: 1.42%; 6 months: 2.31%; 9 months: 3.12%; 12 months: 3.92%; all *p* ≤ 0.01).

**FIGURE 2:**
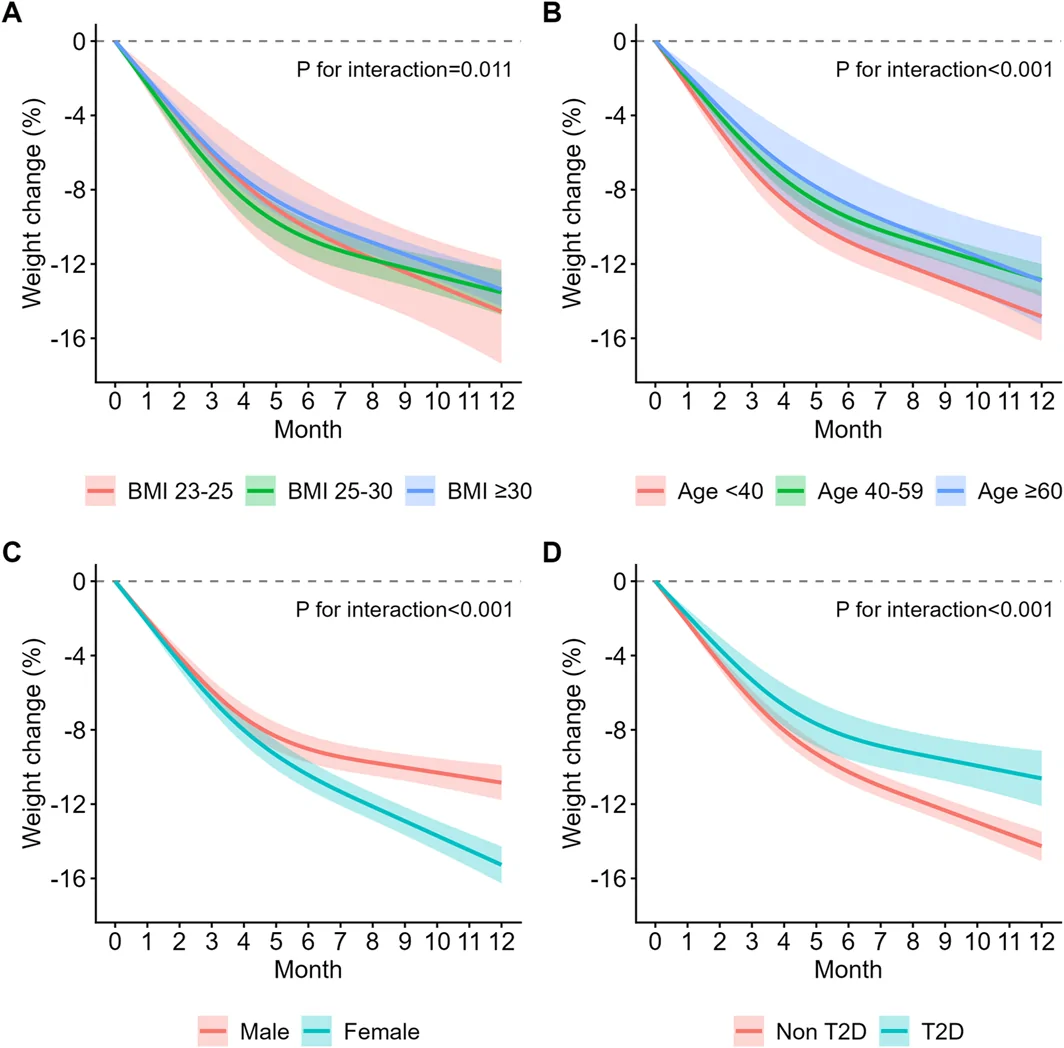
Percentage total body weight reduction over 12 months with semaglutide by baseline subgroup characteristics. Estimated mean percentage changes in body weight from baseline over 12 months are shown by (A) baseline body mass index (BMI) categories (23 to < 25, 25 to < 30, and ≥ 30 kg/m^2^), (B) age groups (< 40, 40–59, and ≥ 60 years), (C) sex, and (D) type 2 diabetes (T2D) status. Data are presented as estimated marginal means with 95% confidence intervals (shaded areas), derived from linear mixed‐effects models for repeated measures. Significant differences in weight‐loss trajectories were observed across subgroups for baseline BMI, age, sex, and T2D status (*p* for interaction indicated in each panel). Overall, greater percentage weight reduction was observed in individuals with younger age, female sex, and those without T2D.

Of 1160 participants, 474 (40.9%) had prior obesity medication exposure, including 290 (25.0%) with prior liraglutide use and 118 (10.2%) with prior phentermine/topiramate use; the remaining 686 (59.1%) were medication‐naïve. Significant reductions in percentage body weight were observed in all three groups (Supporting Information S1: Figure 1), but weight‐loss trajectories differed significantly by prior medication exposure (*p* for interaction < 0.001). Medication‐naïve participants achieved significantly greater weight reduction than those with prior liraglutide or phentermine/topiramate use from 3 months onward (all *p* < 0.001). At 12 months, percentage body weight reduction was −16.3%, −13.8%, and −11.3% in the medication‐naïve, phentermine/topiramate, and liraglutide groups; no significant difference was observed between the two prior‐medication groups.

### 3.4 Changes in Cardiometabolic Risk Factors

Significant improvements in cardiometabolic parameters were observed throughout the follow‐up period (mean: 4.9 months; Supporting Information S1: Table 2). Given the limited sample size at 12 months (*n* = 23), 6‐month data are presented as the primary timepoint, with full results available in Supporting Information S1: Table 2.

SBP and DBP decreased significantly at 6 months (SBP: −10.76 mmHg, 95% CI −11.56 to −9.96; DBP: −5.50 mmHg, 95% CI −6.22 to −4.77; both *p* < 0.001), with reductions apparent from 3 months and sustained through 9 months (all *p* < 0.001).

Fasting glucose and HbA1c were reduced at 6 months (−18.10 mg/dL, 95% CI −19.92 to −16.27; −0.92%, 95% CI −0.99 to −0.85; both *p* < 0.001) and maintained at 9 months (all *p* < 0.001).

Total and low‐density lipoprotein cholesterol levels decreased markedly at 6 months (−24.06 and −15.21 mg/dL; both *p* < 0.001), with attenuated but significant reductions at 9 months (both *p* < 0.001). High‐density lipoprotein cholesterol showed an initial decrease at 3 months, followed by a significant progressive increase from 9 months onward (+3.51 mg/dL; 95% CI 2.13 to 4.89; *p* < 0.001). Triglycerides decreased consistently, reaching −38.79 mg/dL (95% CI −49.20 to −28.38) at 6 months and −41.83 mg/dL (95% CI −57.23 to −26.43) at 9 months (all *p* < 0.001).

Aspartate aminotransferase (AST) and alanine aminotransferase (ALT) improved significantly at 6 months (AST: −11.83 U/L, 95% CI −13.48 to −10.17; ALT: −16.49 U/L, 95% CI −20.35 to −12.64) and 9 months (both *p* < 0.001), indicating sustained hepatic benefit.

### 3.5 Changes in Body Composition

Significant improvements in body composition were observed throughout the follow‐up period (Figure 3). Given the limited sample size at 12 months (*n* = 33), 6‐month data are presented as the primary timepoint, with full results across all timepoints available in Figure 3. Fat mass decreased progressively, with an estimated mean change of −6.26 kg (95% CI, −6.70 to −5.82) at 6 months and −7.84 kg (95% CI, −8.34 to −7.34) at 9 months (all *p* < 0.001). Lean mass also declined to a lesser extent, with reductions of −2.46 kg (95% CI, −2.68 to −2.23) at 6 months and −2.64 kg (95% CI, −2.92 to −2.37) at 9 months (all *p* < 0.001).

**FIGURE 3:**
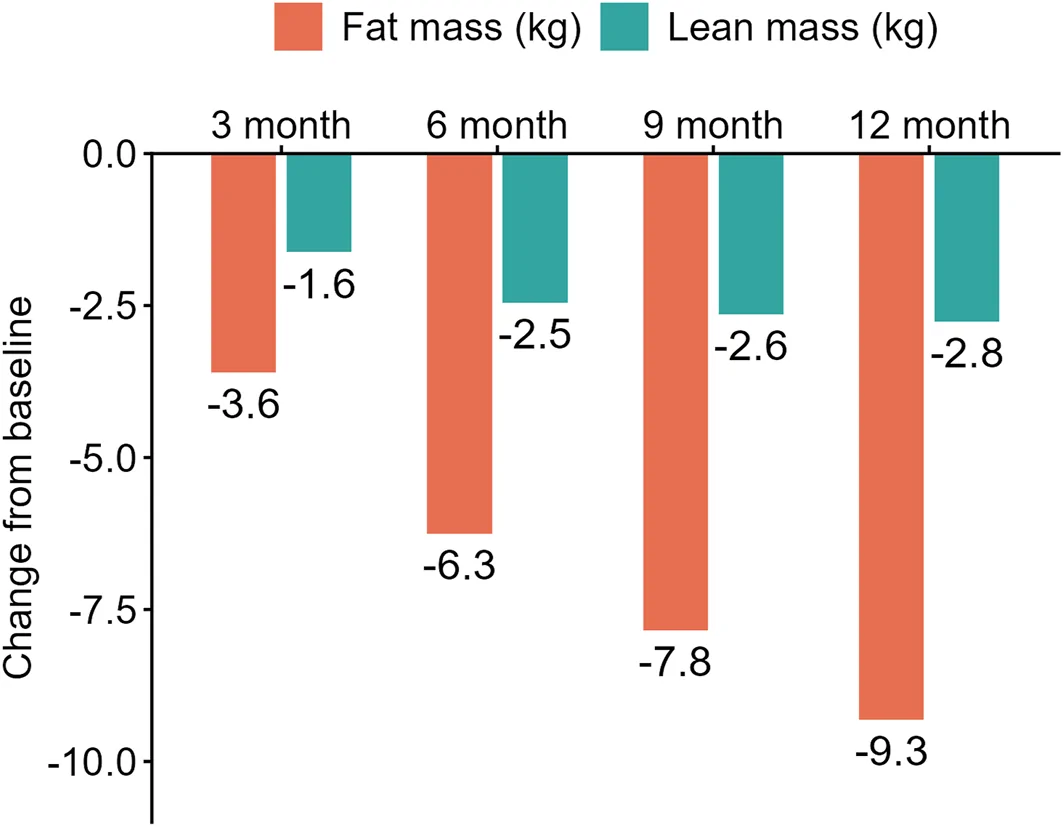
Changes in body composition over 12 months assessed by bioelectrical impedance analysis. Estimated mean changes in fat mass (orange) and lean mass (green) from baseline at 3, 6, 9, and 12 months. Bioelectrical impedance analysis data were available for 548 patients at baseline, 318 at 3 months, 177 at 6 months, 92 at 9 months, and 33 at 12 months.

### 3.6 Treatment Patterns and Persistence

Semaglutide dose distribution over time is summarised in Supporting Information S1: Table 3. Most patients initiated at the recommended starting dose of 0.25 mg (55.9%), with the remainder starting at higher doses (0.5 mg, 30.4%; 1.0 mg, 11.9%), reflecting individualised dosing in real‐world practice. Dose escalation proceeded rapidly thereafter, with the proportion receiving the maintenance dose of 2.4 mg peaking at 76.1% by month 6 and a mean time to reaching the maintenance dose of 3.52 months.

Treatment persistence declined over time; the proportion of patients who discontinued semaglutide increased from 2.6% at month 1 to 10.6% at month 6, with a mean treatment duration of 4.94 ± 2.76 months. The most common reasons for discontinuation were patient request (25.1%), lack of effectiveness (21.4%), and adverse events (10.4%) (Supporting Information S1: Figure 2).

### 3.7 Safety and Tolerability

Adverse events were reported by approximately 22% of patients (Supporting Information S1: Figure 3). Gastrointestinal symptoms predominated, with nausea being the most common (14.6% of patients at month 1), declining progressively over the treatment course. Other gastrointestinal events, including vomiting, diarrhoea, and abdominal pain, were less frequent and similarly transient.

## 4 Discussion

The RE‐SEMA‐K study provides multicenter real‐world evidence on the effectiveness and safety of once‐weekly semaglutide 2.4 mg in a South Korean population, where semaglutide 2.4 mg was associated with substantial and sustained weight reduction, accompanied by improvements in body composition, and cardiometabolic status.

### 4.1 Effectiveness in the South Korean Context

The magnitude of weight reduction observed in our cohort closely aligns with the STEP 6 trial findings in an East Asian population [6]. Both studies included individuals with T2D, enhancing clinical relevance for mixed populations encountered in routine practice, where patients with and without diabetes are treated concurrently. This is particularly noteworthy given that the mean treatment duration in our cohort was only 4.9 months—substantially shorter than the 68‐week follow‐up of the STEP 6 trial—and achieved comparable weight loss (−13.16% vs. −13.2% in STEP 6), suggesting that clinically meaningful weight reduction is achievable within a substantially shorter period in real‐world practice.

Furthermore, our cohort included a substantial proportion of treatment‐experienced patients, a population not well‐represented in the STEP programme, including those with prior liraglutide use and prior phentermine/topiramate use. This is clinically significant because prior obesity medication exposure is associated with an attenuated treatment response. Despite this high proportion of treatment‐experienced patients, the overall magnitude of weight reduction remained comparable to that reported in STEP 6, suggesting that the effectiveness of semaglutide 2.4 mg extends beyond treatment‐naïve populations and can be maintained in patients transitioning from previous pharmacologic obesity therapies.

Our findings were also comparable to Western real‐world data, including the US‐based SCOPE study (−10.0% at 6 months; mean BMI 37.9 kg/m^2^), despite our cohort having substantially lower baseline BMI (32.4 kg/m^2^), suggesting effectiveness across a broader spectrum of obesity severity [17]. A substantial proportion of patients achieved ≥ 5% and ≥ 10% weight loss—thresholds associated with clinically meaningful cardiometabolic improvements—further underscoring the real‐world relevance of semaglutide even at lower baseline BMI [18].

Subgroup analyses revealed differential treatment responses across baseline characteristics, with implications for individualised obesity management.

Notably, percentage body weight reduction was consistent across all BMI categories, indicating that the relative efficacy is independent of initial obesity severity. However, patients without T2D achieved greater percentage weight reduction than those with T2D, aligned with previous GLP‐1 receptor agonist trials demonstrating attenuated response in individuals with T2D [5, 9, 10]. Modestly greater weight loss was also observed in younger individuals and women, consistent with prior reports suggesting hormonal, behavioural, and metabolic influences on treatment response [18, 19]. Improvements in cardiometabolic risk factors accompanied weight reduction in this cohort, broadly consistent with effects reported with other GLP‐1 receptor agonists. However, given the observational design of this study, these findings should be interpreted as descriptive, and causal inference regarding an independent metabolic benefit beyond weight loss itself cannot be established.

### 4.2 Body Composition and Lean Mass Preservation

A critical consideration in pharmacologic weight loss is the relative reduction in lean mass [20]. In our study, ∼71% of the weight loss at 6 months was attributable to fat mass, corresponding to a fat‐to‐lean mass loss ratio of approximately 2.5:1. These findings are consistent with those from clinical trials, including the STEP 1 body composition sub‐study, which reported that ∼70% of weight loss was derived from fat mass [5]. Preferential fat mass reduction is particularly relevant in Asian populations, including South Koreans, who are predisposed to increased visceral adiposity even at lower BMI levels [21]. This pattern of body composition change suggests a metabolically favourable profile of weight loss [22, 23]. However, the observed reduction in lean mass underscores the need to monitor body composition and consider strategies for preserving skeletal muscle during treatment.

### 4.3 Real‐World Persistence and Barriers

Treatment persistence in our cohort was lower than that reported in clinical trials, reflecting real‐world challenges including limited adherence support, patient expectations, and the substantial out‐of‐pocket cost resulting from the lack of insurance reimbursement for obesity medications in South Korea [24]. Furthermore, in routine practice, some patients discontinue treatment upon achieving a self‐defined target weight rather than continuing long‐term maintenance therapy as clinical guidelines recommend [25]. Notably, lack of efficacy accounted for 21.4% of discontinuations, suggesting that ‘slow responders’ without meaningful early weight loss may be reluctant to continue paying for expensive self‐funded therapy, in contrast to clinical trials with protocol‐mandated adherence and free medication [26].

Dose escalation was also notably rapid in our cohort (mean time to maintenance dose: 3.52 months vs. the recommended 16‐week titration schedule). This acceleration was likely driven by the high proportion of treatment‐experienced individuals (40.9%), and the uniform pricing structure across all pen doses in South Korea until July 2025, which financially incentivised faster escalation. These observations illustrate how clinical history and economic factors interact to shape prescribing patterns in a real‐world, non‐reimbursed setting; flexible dosing strategies may ultimately be needed to optimise long‐term adherence.

### 4.4 Strengths and Limitations

The RE‐SEMA‐K study has several strengths. First, it employed a multicenter design with a large sample size across 17 institutions in South Korea, enhancing generalisability to routine practice. Second, comprehensive treatment pattern data—including subgroup analyses and discontinuation reasons—offer insights into real‐world effectiveness and persistence in heterogeneous populations. Several limitations should be acknowledged. The retrospective design introduces the potential for residual confounding and selection bias. Additionally, missing data for certain variables—particularly lifestyle factors and body composition measures—may limit the interpretability of some secondary outcomes. In particular, given the substantial proportion of missing data for several cardiometabolic and hepatic parameters, these secondary findings should be interpreted as exploratory rather than confirmatory. Furthermore, the declining patient numbers at later follow‐up time points may affect the precision of long‐term estimates. Future studies are warranted to identify strategies for improving long‐term treatment persistence and evaluate optimal maintenance approaches, including the potential role of lower or individualised dosing strategies in sustaining weight loss.

## 5 Conclusions

We show that once‐weekly semaglutide 2.4 mg is effective and generally safe for South Korean adults with overweight or obesity. In real‐world settings encompassing patients with and without T2D, it induced significant weight loss and marked improvement in metabolic health, achieving outcomes comparable to those reported in STEP 6 despite substantial prior obesity medication exposure and relatively short treatment persistence. Addressing financial barriers and optimising long‐term treatment adherence will be important to maximise its clinical impact and long‐term cardiometabolic benefits.

## Author Contributions

Yu‐Jin Kwon contributed to the study design, data collection, data interpretation, and manuscript drafting. Jee‐Hyun Kang contributed to the study design, data collection, funding acquisition, overall supervision, and critical revision of the manuscript. Kyoung‐Kon Kim contributed to the study design, data collection, data interpretation, manuscript drafting, critical revision, and overall supervision. Seok‐Jae Heo was responsible for data analysis. All other authors (Young‐Sang Kim, Hae‐Jin Ko, Susie Jung, Yoon Jeong Cho, Ji‐Eun Song, Sung‐Bum Lee, Ga Eun Nam, Jungha Park, Myung Jae Seo, Jong Koo Kim, Seo Young Kang, Yeong Sook Yoon, Soo Hyun Cho, Yonghwan Kim, Hye‐ji An, Seung Hee Kim, Yoo‐Bin Seo, and Yong Ho Lee) contributed to data collection and critical review of the manuscript. Jee‐Hyun Kang and Kyoung‐Kon Kim served as corresponding authors. All authors reviewed and approved the final manuscript.

## Funding

This research was supported by the Society for Korean Obesity and Metabolism Studies.

## Conflicts of Interest

Dr. Yu‐Jin Kwon reports lecture fees from Alvogen Korea, Chong Kun Dang, Eli Lilly Korea, and Novo Nordisk Pharma Korea. Dr. Young‐Sang Kim reports lecture fees from Novo Nordisk Pharma Korea and Eli Lilly Korea. Dr. Hae‐Jin Ko reports research grants from Novo Nordisk, Boehringer Ingelheim, Huons, Hanmi Pharmaceutical, HK inno.N, Amgen, and Eli Lilly; and lecture fees from Alvogen Korea, Chong Kun Dang, Eli Lilly Korea, and Novo Nordisk Pharma Korea. Dr. Susie Jung, Dr. Yoon Jeong Cho, and Dr. Ji‐Eun Song report lecture fees from Alvogen Korea, Chong Kun Dang, Eli Lilly Korea, and Novo Nordisk Pharma Korea. Dr. Sung‐Bum Lee has no conflicts of interest to declare. Dr. Ga Eun Nam, Dr. Jungha Park, and Dr. Myung Jae Seo report lecture fees from Alvogen Korea, Chong Kun Dang, Eli Lilly Korea, and Novo Nordisk Pharma Korea. Dr. Jong Koo Kim has no conflicts of interest to declare. Dr. Seo Young Kang and Dr. Yeong Sook Yoon report lecture fees from Alvogen Korea, Chong Kun Dang, Eli Lilly Korea, and Novo Nordisk Pharma Korea. Dr. Soo Hyun Cho has no conflicts of interest to declare. Dr. Yonghwan Kim reports lecture fees from Novo Nordisk Pharma Korea and Eli Lilly Korea. Dr. Hye‐ji An, Dr. Seung Hee Kim, and Dr. Yoo‐Bin Seo report lecture fees from Alvogen Korea, Chong Kun Dang, Eli Lilly Korea, and Novo Nordisk Pharma Korea. Dr. Yong Ho Lee and Dr. Seok‐Jae Heo have no conflicts of interest to declare. Dr. Jee‐Hyun Kang reports research grants from Chong Kun Dang, Hanmi Pharmaceutical, Amgen, HK inno.N, Eli Lilly, and Novo Nordisk Pharma; consulting fees for Eli Lilly Korea, Boehringer Ingelheim, and Novo Nordisk Pharma Korea; and honoraria from Alvogen Korea, Chong Kun Dang, Eli Lilly Korea, and Novo Nordisk Pharma Korea. Dr. Kyoung‐Kon Kim reports clinical trial contracts from Hanmi Pharmaceutical, Alvogen Korea, Huons, Amgen, Boehringer Ingelheim, AstraZeneca, Eli Lilly, and Novo Nordisk Pharma; consulting fees for Eli Lilly Korea and Novo Nordisk Pharma Korea; and honoraria from Hanmi Pharmaceutical, Alvogen Korea, Chong Kun Dang, Eli Lilly Korea, and Novo Nordisk Pharma Korea.

## Supporting information


Supporting Information S1

## Data Availability

The data that support the findings of this study are available from the corresponding author upon reasonable request.
