# Supplementary material for: Effectiveness and Safety of Once‐Weekly Semaglutide 2.4 mg in Korean Adults With Obesity or Overweight: A Multicenter Retrospective Real‐World Study (RE‐SEMA‐K Study)
Source: Diabetes Metab Res Rev. 2026 Nov 9;42(8):e70242. doi: 10.1002/dmrr.70242 (PMC13746800; doi:10.1002/dmrr.70242)
Supplement: Supplementary file 1 [file DMRR-42-e70242-s001.docx]

**Supporting Information S1: Supplementary Tables and Figures**

**Effectiveness and Safety of Once-Weekly Semaglutide 2.4 mg in Korean Adults With Obesity or Overweight: A Multicenter Retrospective Real-World Study (RE-SEMA-K Study)**

**List of Supporting Information**

**Supplementary Table 1. Proportion of patients achieving clinically meaningful weight loss over time**

**Supplementary Table 2. Changes in cardiometabolic parameters over 12 months following treatment with semaglutide 2.4 mg**

**Supplementary Table 3. Semaglutide dose distribution and treatment status over 12 months**

**Supplementary Figure 1. Percentage total body weight reduction over 12 months with semaglutide by prior obesity medication exposure**

**Supplementary Figure 2. Reasons for discontinuation of treatment with Wegovy (semaglutide) during follow-up**

**Supplementary Figure 3. Gastrointestinal adverse events over the follow-up period following treatment with semaglutide 2.4 mg**

**Supplementary Table 1. Proportion of patients achieving clinically meaningful weight loss over time**

| Weight loss threshold | Month | Proportion (%) (95% CI) | P value |
| --- | --- | --- | --- |
| ≥5% weight loss | 3 | 53.82 (51.95–55.67) | <0.001 |
|  | 6 | 78.09 (76.47–79.62) | <0.001 |
|  | 9 | 76.02 (73.16–78.67) | <0.001 |
|  | 12 | 71.78 (65.72–77.15) | <0.001 |
| ≥10% weight loss | 3 | 14.58 (13.24–16.03) | <0.001 |
|  | 6 | 50.03 (47.72–52.35) | <0.001 |
|  | 9 | 52.62 (49.34–55.88) | <0.001 |
|  | 12 | 51.72 (45.19–58.21) | <0.001 |
| ≥15% weight loss | 3 | 3.00 (2.38–3.78) | <0.001 |
|  | 6 | 18.91 (16.97–21.02) | <0.001 |
|  | 9 | 25.01 (22.29–27.93) | <0.001 |
|  | 12 | 29.32 (23.25–36.22) | <0.001 |
| ≥20% weight loss | 3 | 0.68 (0.42–1.10) | <0.001 |
|  | 6 | 5.36 (4.29–6.67) | <0.001 |
|  | 9 | 9.49 (7.79–11.51) | <0.001 |
|  | 12 | 14.62 (9.80–21.26) | <0.001 |

Data are presented as estimated proportions with 95% confidence intervals (CIs) derived from linear mixed-effects models for repeated measures. P values represent weight comparisons versus baseline. Missing data were assumed to occur at random.

**Supplementary Table 2. Changes in cardiometabolic parameters over 12 months following treatment with semaglutide 2.4 mg**

| Parameter | Month | Estimated marginal mean (95% CI) | P value |
| --- | --- | --- | --- |
| SBP (mmHg) | 3 | -8.69 (-9.42, -7.95) | <0.001 |
|  | 6 | -10.76 (-11.56, -9.96) | <0.001 |
|  | 9 | -10.63 (-11.69, -9.58) | <0.001 |
|  | 12 | -10.36 (-12.02, -8.69) | <0.001 |
| DBP (mmHg) | 3 | -4.45 (-5.13, -3.78) | <0.001 |
|  | 6 | -5.50 (-6.22, -4.77) | <0.001 |
|  | 9 | -5.64 (-6.55, -4.73) | <0.001 |
|  | 12 | -5.73 (-7.10, -4.35) | <0.001 |
| Glucose (mg/dL) | 3 | -17.09 (-18.76, -15.41) | <0.001 |
|  | 6 | -18.10 (-19.92, -16.27) | <0.001 |
|  | 9 | -16.94 (-19.48, -14.39) | <0.001 |
|  | 12 | -15.49 (-20.17, -10.82) | <0.001 |
| HbA1c (%) | 3 | -0.63 (-0.70, -0.57) | <0.001 |
|  | 6 | -0.92 (-0.99, -0.85) | <0.001 |
|  | 9 | -0.95 (-1.05, -0.86) | <0.001 |
|  | 12 | -0.94 (-1.12, -0.76) | <0.001 |
| TC (mg/dL) | 3 | -24.76 (-28.88, -20.65) | <0.001 |
|  | 6 | -24.06 (-28.65, -19.46) | <0.001 |
|  | 9 | -17.23 (-23.38, -11.08) | <0.001 |
|  | 12 | -9.60 (-20.46, 1.26) | 0.083 |
| LDL (mg/dL) | 3 | -18.49 (-22.17, -14.81) | <0.001 |
|  | 6 | -15.21 (-19.19, -11.22) | <0.001 |
|  | 9 | -11.07 (-16.44, -5.70) | <0.001 |
|  | 12 | -6.80 (-16.96, 3.35) | 0.189 |
| HDL (mg/dL) | 3 | -2.63 (-3.62, -1.64) | <0.001 |
|  | 6 | -0.88 (-1.94, 0.19) | 0.105 |
|  | 9 | 3.51 (2.13, 4.89) | <0.001 |
|  | 12 | 8.30 (5.80, 10.81) | <0.001 |
| TG (mg/dL) | 3 | -34.82 (-43.90, -25.73) | <0.001 |
|  | 6 | -38.79 (-49.20, -28.38) | <0.001 |
|  | 9 | -41.83 (-57.23, -26.43) | <0.001 |
|  | 12 | -44.74 (-74.05, -15.43) | 0.003 |
| AST (U/L) | 3 | -8.60 (-10.10, -7.09) | <0.001 |
|  | 6 | -11.83 (-13.48, -10.17) | <0.001 |
|  | 9 | -11.88 (-14.25, -9.51) | <0.001 |
|  | 12 | -11.51 (-15.93, -7.09) | <0.001 |
| ALT (U/L) | 3 | -13.02 (-16.55, -9.48) | <0.001 |
|  | 6 | -16.49 (-20.35, -12.64) | <0.001 |
|  | 9 | -14.79 (-20.19, -9.38) | <0.001 |
|  | 12 | -12.40 (-22.32, -2.47) | 0.014 |

Data are presented as estimated marginal means with 95% confidence intervals derived from linear mixed-effects models for repeated measures. Changes from baseline are shown at 3, 6, 9, and 12 months. Negative values indicate reductions from baseline, whereas positive values indicate increases. Analyses were restricted to time points with at least 15 observations to ensure stability of estimates.

The number of participants with available data decreased over time (e.g., body weight: baseline, n = 1,160; 3 months, n = 858; 6 months, n = 422; 9 months, n = 147; 12 months, n = 23).

ALT, alanine aminotransferase; AST, aspartate aminotransferase; CI, confidence interval; DBP, diastolic blood pressure; HbA1c, glycated hemoglobin; HDL, high-density lipoprotein cholesterol; LDL, low-density lipoprotein cholesterol; SBP, systolic blood pressure; TC, total cholesterol; TG, triglycerides

**Supplementary Table 3. Semaglutide dose distribution and treatment status over 12 months**

| Months | 0M | 1M | 2M | 3M | 4M | 5M | 6M | 7M | 8M | 9M | 10M | 11M | 12M |
| --- | --- | --- | --- | --- | --- | --- | --- | --- | --- | --- | --- | --- | --- |
| N (%) | 1,160 | 758 | 860 | 775 | 756 | 581 | 445 | 420 | 274 | 221 | 156 | 125 | 67 |
| 0.25 mg | 651 (56.1%) | 9 (1.2%) | 2 (0.2%) | 1 (0.1%) | 2 (0.3%) | 1 (0.2%) | 1 (0.2%) | 1 (0.2%) | 1 (0.4%) | 0 (0.0%) | 1 (0.6%) | 1 (0.8%) | 0 (0.0%) |
| 0.5 mg | 354 (30.5%) | 391 (51.6%) | 96 (11.2%) | 14 (1.8%) | 10 (1.3%) | 6 (1.0%) | 10 (2.2%) | 8 (1.9%) | 6 (2.2%) | 2 (0.9%) | 4 (2.6%) | 3 (2.4%) | 2 (3.0%) |
| 1.0 mg | 136 (11.7%) | 238 (31.4%) | 403 (46.9%) | 158 (20.4%) | 53 (7.0%) | 25 (4.3%) | 20 (4.5%) | 28 (6.7%) | 15 (5.5%) | 11 (5.0%) | 8 (5.1%) | 4 (3.2%) | 6 (9.0%) |
| 1.7 mg | 13 (1.1%) | 85 (11.2%) | 234 (27.2%) | 338 (43.6%) | 178 (23.5%) | 65 (11.2%) | 26 (5.8%) | 24 (5.7%) | 22 (8.0%) | 13 (5.9%) | 17 (10.9%) | 12 (9.6%) | 8 (11.9%) |
| 2.4 mg | 5 (0.4%) | 14 (1.8%) | 75 (8.7%) | 224 (28.9%) | 460 (60.8%) | 437 (75.2%) | 332 (74.6%) | 283 (67.4%) | 191 (69.7%) | 144 (65.2%) | 102 (65.4%) | 70 (56.0%) | 37 (55.2%) |
| Discontinued | 0 (0.0%) | 20 (2.6%) | 48 (5.6%) | 39 (5.0%) | 50 (6.6%) | 45 (7.7%) | 46 (10.3%) | 68 (16.2%) | 32 (11.7%) | 43 (19.5%) | 23 (14.7%) | 30 (24.0%) | 10 (14.9%) |
| Others | 1 (0.1%) | 1 (0.1%) | 2 (0.2%) | 1 (0.1%) | 3 (0.4%) | 2 (0.3%) | 10 (2.2%) | 8 (1.9%) | 7 (2.6%) | 8 (3.6%) | 1 (0.6%) | 5 (4.0%) | 4 (6.0%) |

Values are presented as n (%). Percentages were calculated using the number of patients with available follow-up data at each time point as the denominator. Follow-up time is expressed in months after semaglutide initiation. Dose categories represent the prescribed once-weekly semaglutide dose at each follow-up assessment. “Discontinued” indicates patients who had stopped semaglutide treatment before the corresponding follow-up assessment. “Others” indicates patients who self-adjusted their dose.

**Supplementary Figure 1. Percentage total body weight reduction over 12 months with semaglutide** **by prior obesity medication exposure**

Estimated mean percentage change in body weight from baseline over time according to prior obesity medication use (liraglutide, phentermine/topiramate, or none).

Data are presented as estimated marginal means with 95% confidence intervals (shaded area) derived from linear mixed-effects models for repeated measures (P for interaction <0.001).
**Supplementary Figure 2. Reasons for discontinuation of treatment with Wegovy (semaglutide) during follow-up**

The pie chart shows the distribution of reasons for treatment discontinuation among patients who discontinued semaglutide 2.4 mg during the study period. The most common reasons for discontinuation were other reasons (25.7%), patient request (25.1%), and lack of effectiveness (21.4%), followed by adverse events (10.4%), achievement of target weight (7.5%), investigator decision (6.1%), and cost burden (3.9%).

**Supplementary Figure 3. Gastrointestinal adverse events over the follow-up period following treatment with semaglutide 2.4 mg**

Percentage of patients reporting gastrointestinal adverse events (nausea, vomiting, diarrhea, constipation, and abdominal pain) at 1, 3, 6, and 9 months after treatment initiation.
